# The Number and Complexity of Pure and Recombinant HIV-1 Strains Observed within Incident Infections during the HIV and Malaria Cohort Study Conducted in Kericho, Kenya, from 2003 to 2006

**DOI:** 10.1371/journal.pone.0135124

**Published:** 2015-08-19

**Authors:** Erik Billings, Eric Sanders-Buell, Meera Bose, Andrea Bradfield, Esther Lei, Gustavo H. Kijak, Miguel A. Arroyo, Rukia M. Kibaya, Paul T. Scott, Monique K. Wasunna, Frederick K. Sawe, Douglas N. Shaffer, Deborah L. Birx, Francine E. McCutchan, Nelson L. Michael, Merlin L. Robb, Jerome H. Kim, Sodsai Tovanabutra

**Affiliations:** 1 United States Military HIV Research Program/Henry M. Jackson Foundation, Rockville, Maryland, United States of America; 2 United States Military HIV Research Program/Walter Reed Army Institute of Research, Silver Spring, Maryland, United States of America; 3 The Kenya Medical Research Institute/Walter Reed Project Clinical Research Center, Kericho, Kenya; 4 The Kenya Medical Research Institute, Kericho, Kenya; 5 The Kenya Medical Research Institute, Nairobi, Kenya; 6 The Kenya Medical Research Institute/Walter Reed Project HIV Program, Kericho, Kenya; 7 United States Army Medical Research Unit-Kenya/Walter Reed Project HIV Program, Kericho, Kenya; 8 International Vaccine Institute, Seoul, Republic of Korea; Institut Pasteur of Shanghai,Chinese Academy of Sciences, CHINA

## Abstract

Characterization of HIV-1 subtype diversity in regions where vaccine trials are conducted is critical for vaccine development and testing. This study describes the molecular epidemiology of HIV-1 within a tea-plantation community cohort in Kericho, Kenya. Sixty-three incident infections were ascertained in the HIV and Malaria Cohort Study conducted in Kericho from 2003 to 2006. HIV-1 strains from 58 of those individuals were full genome characterized and compared to two previous Kenyan studies describing 41 prevalent infections from a blood bank survey (1999–2000) and 21 infections from a higher-risk cohort containing a mix of incident and prevalent infections (2006). Among the 58 strains from the community cohort, 43.1% were pure subtypes (36.2% A1, 5.2% C, and 1.7% G) and 56.9% were inter-subtype recombinants (29.3% A1D, 8.6% A1CD, 6.9% A1A2D, 5.2% A1C, 3.4% A1A2CD, and 3.4% A2D). This diversity and the resulting genetic distance between the observed strains will need to be addressed when vaccine immunogens are chosen. In consideration of current vaccine development efforts, the strains from these three studies were compared to five candidate vaccines (each of which are viral vectored, carrying inserts corresponding to parts of *gag*, *pol*, and *envelope*), which have been developed for possible use in sub-Saharan Africa. The sequence comparison between the observed strains and the candidate vaccines indicates that in the presence of diverse recombinants, a bivalent vaccine is more likely to provide T-cell epitope coverage than monovalent vaccines even when the inserts of the bivalent vaccine are not subtype-matched to the local epidemic.

## Introduction

The Kenya AIDS Response Progress Report estimates a 2012 HIV-1 prevalence of 5.6% among men and women between the ages of 15 to 49 years old [1]. This represents a decrease from previous rates of 6.8% in 2003, 7.6% in 2007, and 6.4% in 2008, and may be part of an overall stabilization of HIV-1 prevalence in Kenya. The estimated number of new infections has hovered around 100,000 per year for 2012 and 2013. Over 78% of transmissions are heterosexual in nature, most commonly among young adults between 15 to 24 years old and mostly amongst the females of that age group [2]. In 2013, the number of people estimated to be living with HIV-1 in Kenya was approximately 1.6 million; and the epidemic is considered to be both endemic within the general population as well as concentrated within a few high-risk groups (MSM, FSW, and IDU’s) [1] [3] [4]. Despite the progress attributable to funding of programs in prevention, care and treatment, it is widely acknowledged that an efficacious vaccine would be the most cost-effective means to control the spread of HIV-1 [5] [6].

Viral diversity is considered to be one of the main challenges facing vaccine development [7]. In the global HIV-1 epidemic, diversity within subtypes in different geographic regions and at-risk populations has been driven by high rates of genetic mutation and recombination, which is a confounding characteristic of retroviruses. The current molecular complexity of the HIV-1 epidemic in Africa is the result of co-circulation of different subtypes in the same geographic area. With respect to Kenya and the surrounding regions: subtypes A1, C, and D co-circulate in Kenya, Uganda, and Tanzania. Subtype A1 is proportionately the largest pure subtype in Kenya with recombinants between A1 and D forming the next largest fraction of infections [8]. In Uganda, subtypes D and A1 make substantial contributions to the epidemic as both pure subtypes and recombinants [9] [10] [11]. In Tanzania, subtype C predominates and co-circulates with subtypes A1, D, and recombinants between the three subtypes [12]. Subtype C strains are also the largest fraction in Ethiopia and South Africa [13] [14].

In an effort to characterize HIV-1 subtype diversity within a segment of Kenyan society considered to be at community-level risk of infection, the HIV and Malaria Cohort Study was conducted to observe prevalent and incident infections within a community cohort of Tea plantation workers near Kericho, Kenya. The initial phase of the study detected 401 prevalent infections from participants infected prior to 2003 [15]. Using the multi-region hybridization assay, it was revealed that HIV-1 subtype A represented the majority (56%) of circulating pure subtypes within the prevalent infections of this cohort, followed by subtypes D (10%) and C (5%); the remaining strains were recombinants (29%) [15]. The present study describes the subsequent incident phase, wherein HIV-1 negative participants were followed from 2003–2006 in order to identify and characterize incident and early infections [16]. Ultimately, 58 full-length HIV-1 genomes were obtained and phylogenetically compared to the full-length sequences obtained during two previous Kenyan studies observing blood-bank samples [8] and a higher-risk cohort [17].

Regarding vaccine development, there are five vectored vaccines currently under development for possible use in East Africa. Four are modified Vaccinia Ankara (MVA) vectored vaccines designed to carry inserts that will express parts of the *gag*, *pol*, and *envelope* genes from HIV-1. Those four vaccines correspond to three regionally tailored variants [18]: MVA-KEA (subtype A1 from Kenya), MVA-TZC (subtype C from Tanzania), MVA-CMDR (CRF01_AE from Thailand); and the bivalent MVA-mosaic which simultaneously delivers two inserts (from subtypes B and C) designed to provide optimal T-cell epitope coverage for a broader global range of subtypes [19,20]. The fifth vaccine uses adenovirus type 26 (Ad26) as the vector and contains mosaic inserts matched with the MVA-mosaic. Recent non-human primate studies have shown that an analogous MVA/Ad26 mosaic SIV vaccine (containing *gag*/*pol*/*env*) was able to produce envelope antibody responses that correlate with protection from SIV acquisition during repetitive low dose mucosal challenges [21] and Gag specific T-cell responses that contributed to enhanced viral load control within the monkeys that eventually became infected after several challenges [22]. Similarly, the Ad26- and MVA-vectored mosaic vaccines intended for use in humans showed an 87–90% reduction in the relative risk of infection against neutralization resistant SHIV162P3 in a low dose intrarectal SHIV challenge model [23]. Those results suggest that MVA or Ad26 vectored HIV-1 vaccines may have therapeutic as well as prophylactic applications. The following work describes the molecular epidemiology of the incident infection strains from this study and the results of a comparative analysis between the candidate HIV-1 vaccines and the observed strains from this study, the blood-bank survey, and the higher-risk cohort.

## Methods

### Study volunteers

In June 2003, the HIV and Malaria Cohort Study among Plantation Workers and Adult Dependents in Kericho, Kenya was initiated. This study was a closed, prospective and community-based cohort of 2,801 volunteers. The protocol was approved by the National Ethical Review Committee under the Kenya Medical Research Institute (KEMRI) and the Walter Reed Army Institute of Research Institutional Review Board. Participants voluntarily provided written, informed consent prior to enrollment in the study. Of the 2,801 volunteers, 401 were excluded after testing seropositive for HIV-1 at the entry examination and the remaining 2,400 seronegative individuals were followed every 6 months for 3 years. Sixty-three incident infections were identified by the end of the study in December 2006. Subject recruitment, counseling, laboratory testing, study methods and results regarding HIV-1 diagnostics, prevalence, incidence, circumcision, local laboratory reference ranges, and HIV-1 genetic diversity and epidemiology have been previously published [15,16,24–27]. The plasma samples collected during this study were used as specimen source for sequencing.

### Laboratory procedures

HIV-1 subtype characterization was performed by full-length genome sequencing of HIV-1 RNA extracted from plasma using the QIAamp Viral RNA Mini Kit. Complementary DNA (cDNA) was synthesized as the complete genome or as two half genomes overlapping by 1.5 kb, using ThermoScript RT (Invitrogen Corp., Carlsbad, CA) as instructed by the manufacturer. Either primer JL68R (5’-CTTCTTCCTGCCATAGGAGATGCCTAAG-3’) or UNINEF-7’ (5’-GCACTCAAGGCAAGCTTTATTGAGGCTT-3’) was used as the 3’ primer to synthesize cDNA. With near-endpoint dilution of cDNA template, a full genome nested PCR was performed. MSF12b/UNINEF-7’ and GAG763 (5’- TGACTAGCGGAGGCTAGAAGGAGAGA-3’)/ TATANEF (5’-GCAGCTGCTTATATGCAGGATCTGAGGG-3’) were the primers used for full genome amplification. PCR products were purified and sequenced by an ABI 3100 capillary sequencer. DNA sequences were assembled using Sequencher version 4.7 and aligned with reference strains from the Los Alamos HIV-1 Database to generate a multiple sequence alignment. The viral strains were preliminarily genotyped using the NCBI Genotyping tool [28].

### Phylogenetic analysis

Initial alignment of viral sequences was performed using HIVAlign [29] and refined with MEGA version 5 [30]. Neighbor-Joining trees were constructed with DIVEIN [31], using the estimated GTR+I+G model, the sequences of interest, and reference strains to designate viral subtypes. All sequences were subjected to BLAST (hiv.lanl.gov) analysis to search for closely related strains and confirm the presence or absence of previously published recombinant forms, which might indicate the spread of a known circulating recombinant form (CRF) or establish the basis for identifying a new CRF. Informative site analysis and visual inspection was performed to verify parent subtypes and precisely map breakpoints within the final genome structures of inter-subtype recombinants [32,33]. In addition, breakpoint assignments were confirmed using the jpHMM tool [34] at http://jphmm.gobics.de and HIV BLAST (hiv.lanl.gov) analysis of sub-genome segments within the recombinants. Pairwise distances between viral genomes or protein sequences are reported in percent and include the interquartile range (IQR), which describes the scatter among the distances by denoting the 25^th^ and 75^th^ percentile values. The distances and corresponding IQR were determined with the pairwise distance calculator within MEGA version 5 using the K2P model (nt) or Poisson model (aa), 0.5 gamma, pairwise deletion, and bootstrapped for 100 replications. Analysis of pairwise distances and epitope coverage between the vaccines and the observed infection strains was conducted using the amino acid sequences of overlapping regions common to each vaccine. Total and Positional T-cell epitope coverage by the vaccines were calculated using the Epitope Coverage Assessment and Positional Epitope Coverage Assessment tools within the Mosaic Vaccine Tool Suite located at hiv.lanl.gov [35]. Statistical significance of sequence distances between the vaccine inserts and observed strains was calculated with Prism 6 using the one-way ANOVA with Bonferroni’s correction (for pure subtype comparison) and the Kruskal-Wallis test with Dunn’s correction (for recombinant comparison) following-log transformation. Normal distribution of the transformed datasets was confirmed for the pure subtype comparison with the D’Agostino & Pearson omnibus and Shapiro-Wilk normality tests within Prism 6. The distribution of vaccine to recombinant strain pairwise distances did not pass the normality tests regardless of transformation strategy.

### Nucleotide sequences

The 58 HIV-1 sequences observed during this community cohort incident infection study have been submitted to GenBank and are available under accession numbers KT022360-KT022417.

## Results

### Study participants

Detailed epidemiological data on incident rates, socio-demographics, behavioral characteristics, and sexually transmitted infections history of this cohort were described by Shaffer and colleagues [16].

### HIV-1 subtype distribution

**Fig 1:**
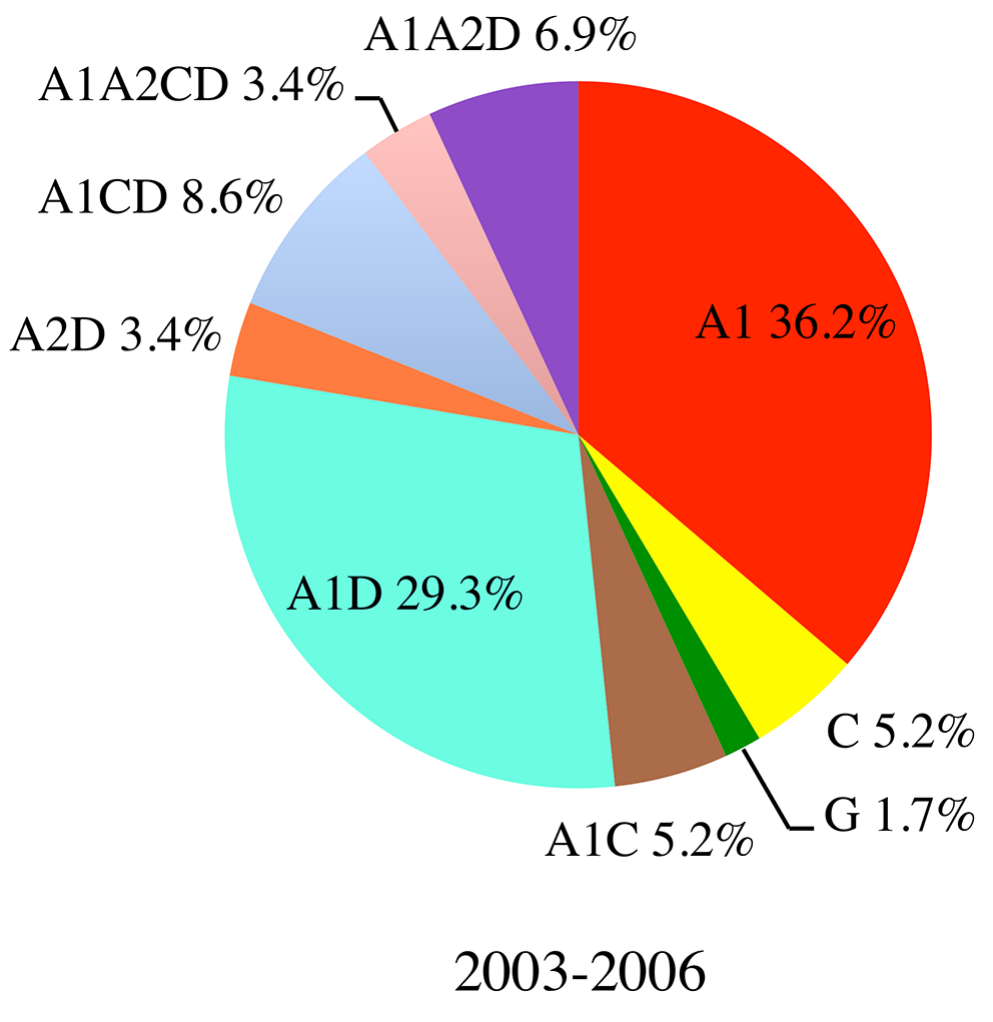
Pure subtype and recombinant virus distribution observed during the incident infection study. The proportion of pure subtype and recombinant strains for the Kericho, Kenya Tea Plantation (community cohort) incident infection study conducted from 2003 to 2006.

From the 63 incident infections identified during the study period, plasma from 62 individuals was available. Of those, 58 were characterized by full genome sequencing. The subtype distribution of the observed sequences is shown in Fig 1. We found that 25 (43.1%) of 58 full genome characterized samples were pure subtypes (36.2% A1, 5.2% C and 1.7% G) and 33 (56.9%) were recombinant forms. The recombinants were between parental subtypes A1, A2, C and D. There were 17 A1D (29.3%), 5 A1CD (8.6%), 4 A1A2D (6.9%), 3 A1C (5.2%), 2 A1A2CD (3.4%), and 2 A2D (3.4%) recombinants. No pure subtype D strains were found in this cohort, but over half of the observed strains (90.9% of the recombinants), contained subtype D genetic material. Likewise, 24.2% of the recombinants contained genetic contributions from the A2 subsubtype, though no pure A2 strains were found.

### Phylogenetic relationships among the pure HIV-1 strains

**Fig 2:**
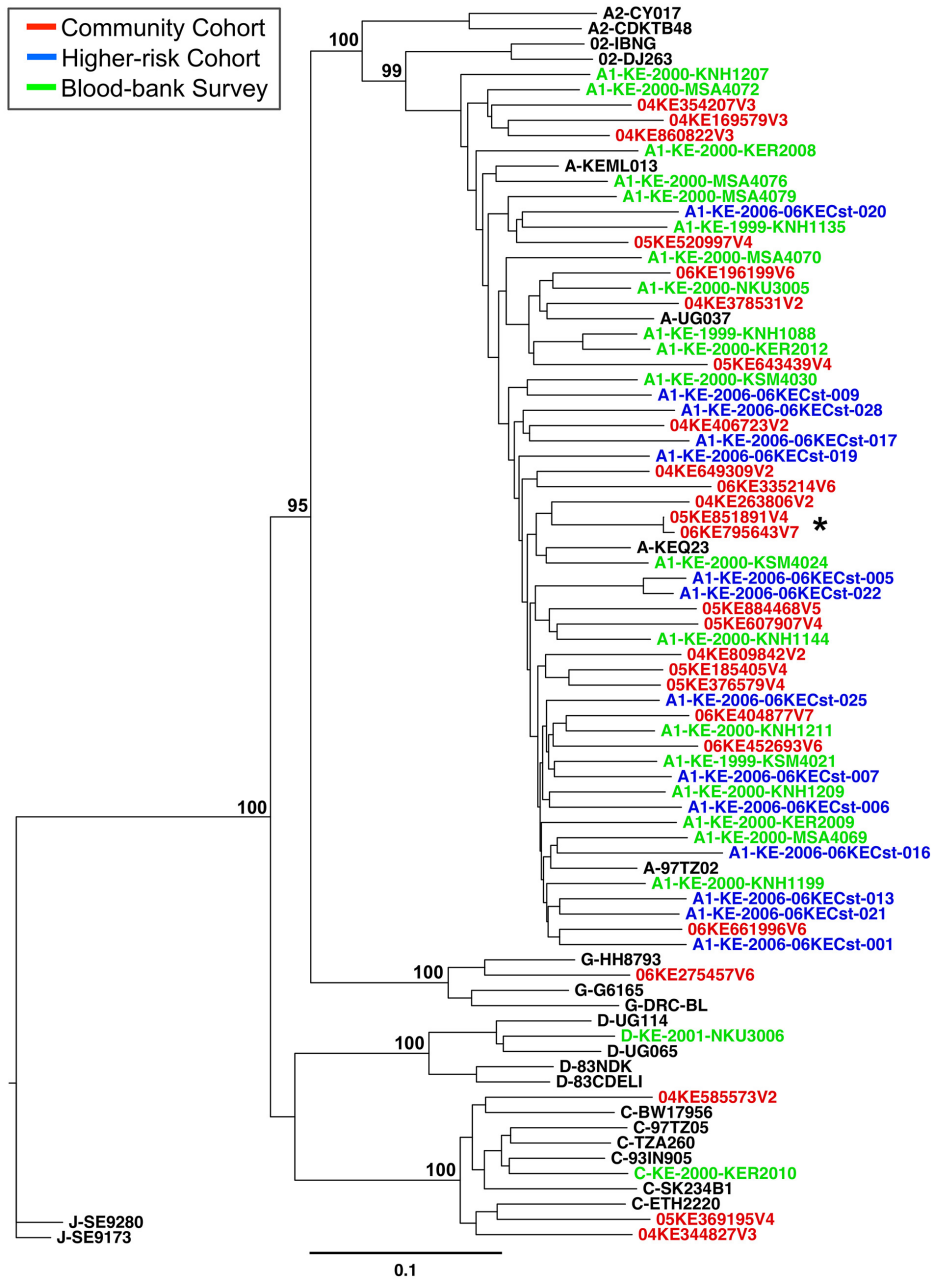
Phylogenetic comparison of pure HIV-1 subtype sequences retrieved from prevalent and incident infections. Full genome sequences from prevalent HIV-1 subtype A1, C, D and G strains previously identified in 1999–2000 (green), a higher-risk (MSM and FSW) cohort identified in 2006 (blue), and the community cohort incident infections identified during this study in 2003–2006 (red), including relevant reference sequences (black) were used to construct a neighbor-joining tree. Bootstrap values at relevant nodes are shown. The scale bar indicates a genetic distance of 10%. Incident infection study participants 05KE851891V4 and 06KE795643V7 (*) are likely linked: pairwise genetic distance of 0.4% (SE 0.06%).

A Neighbor Joining tree of pure subtype strains from the current and previous studies (Fig 2), shows that all pure subtype A1 virus identified in this incident cohort cluster with A1 reference sequences from East Africa. Among the pure A1 community cohort incident strains, 05KE851891V4 and 06KE795643V7 appear to be a directly genetic linked transmission pair with a pairwise distance of 0.4% (SE 0.06%). The demographic characteristics of the participants harboring those two strains show that they were a male and female living in a monogamous civil union; they were from the same ethnicity, same city, and the infection in the male was detected 6 months prior to the female. The subtype A1 sequences from this cohort are interspersed with the prevalent subtype A1 sequences identified during our previous blood bank survey as well as the A1 sequences identified during a more recent study (2006) involving a higher-risk cohort (MSM and FSW) from the Mombasa and Kilifi-Coast areas [17]. The three pure subtype C infections clustered with the single C virus identified during the prevalent infection (blood bank) study and reference subtype C strains from Botswana, Tanzania, Ethiopia, and India. The single subtype G virus (mostly found in West Africa) clustered with subtype G strains previously identified from Kenya [36] with a bootstrap value of 100%. This was confirmed via phylogenetic analysis using over 100 full-genome G and G-containing recombinant sequences (S1 Fig).

### Phylogenetic relationships and genomic structure of inter-subtype recombinants

**Fig 3:**
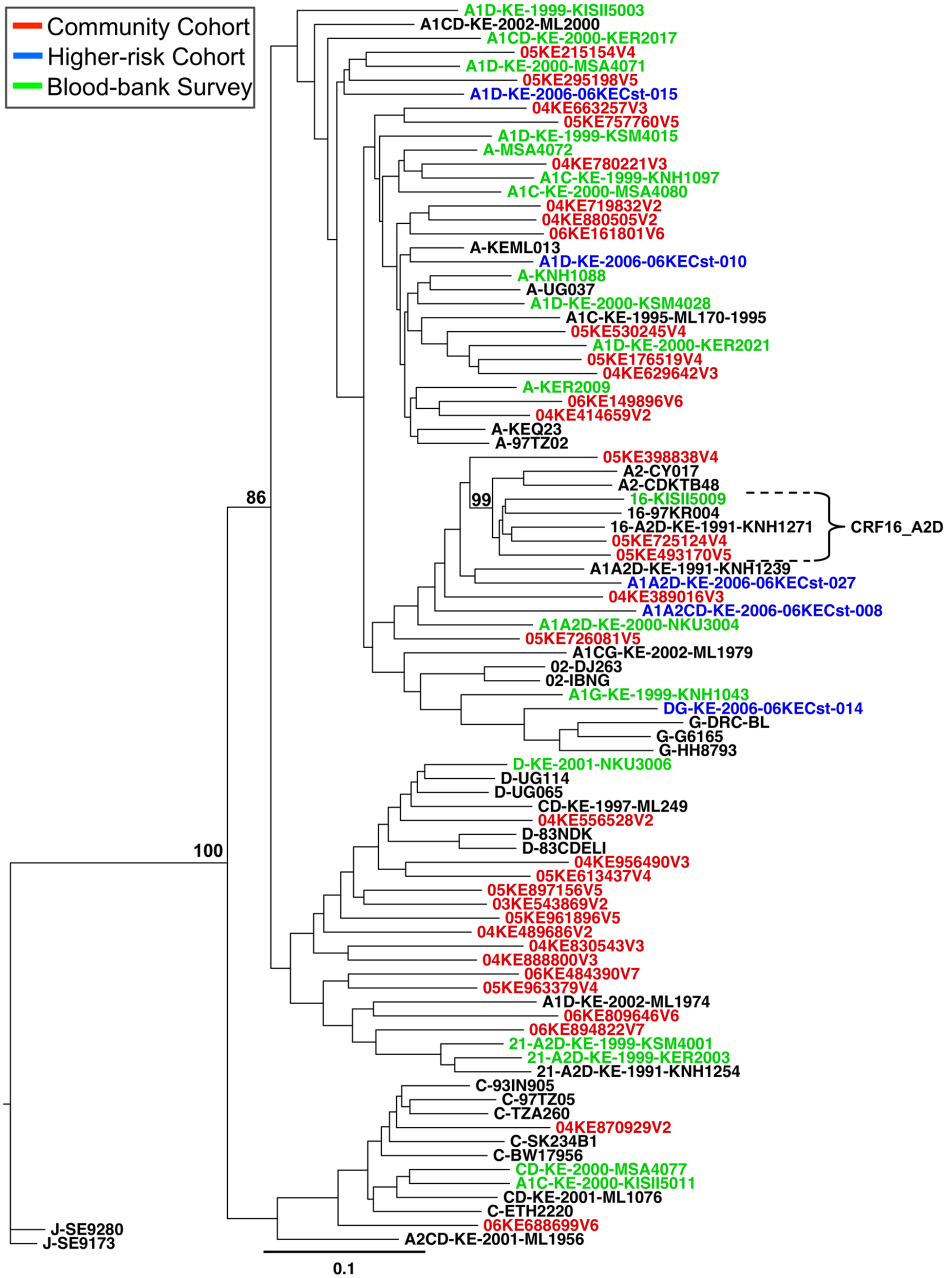
Phylogenetic tree containing the recombinants from the incident, higher-risk, and prevalent HIV-1 infection studies. The phylogenetic relationships between incident recombinant strains from the community cohort (red), higher-risk cohort recombinant strains (blue), and prevalent infection recombinant strains (green) were constructed using the full-length genomes and appropriate pure subtype and recombinant reference sequences. Bootstrap values at relevant nodes are shown. The scale bar indicates a genetic distance of 10%.

**Fig 4:**
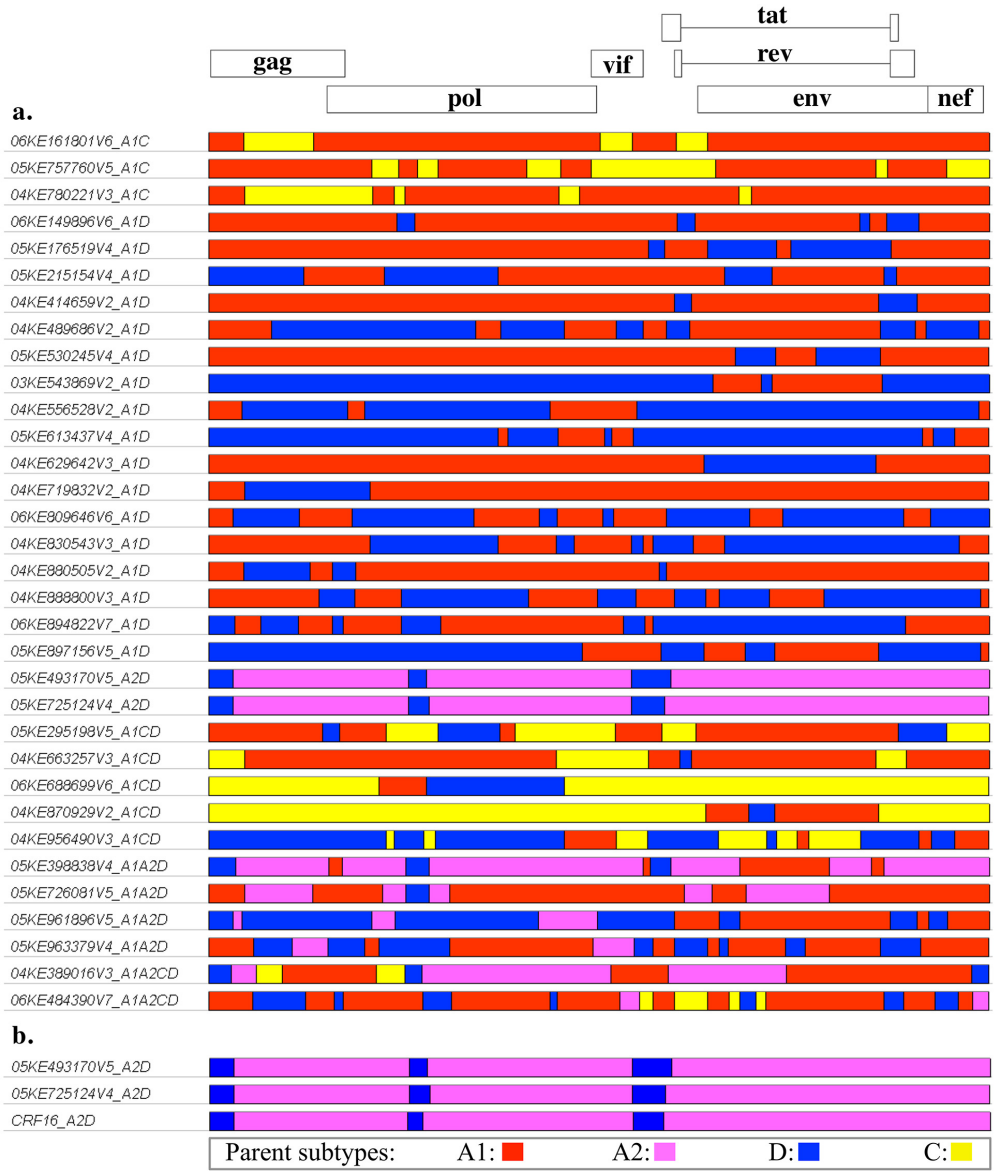
Genome structures of 33 HIV-1 recombinant strains identified during this community cohort incident infection study. (a) The incident recombinant genomes are depicted in relation to the HXB2 reference strain. Each colored region represents the predicted parent subtype based on the results from Recombinant Breakpoint Analysis; subtype A1 is shown in red, A2 in pink, C in yellow, and D in blue. (b) The two A2D strains with similar structures are shown compared to the CRF16_A2D reference breakpoints from hiv.lanl.gov. The A2D strains do appear to be CRF16_A2D infections, but are not directly linked (see text).

A phylogenetic tree of recombinant strains from the current and previous studies (Fig 3) shows an interspersed pattern similar to that seen between the pure subtype strains, with large clusters divided between the parent subtypes A1/A2, C, and D. Within the A1/A2 cluster, two A2D strains (05KE493170V5 and 05KE725124V4) are closely related to the CRF16_A2D variant and reference sequences, collectively forming a distinct sub-cluster. As expected, the two previously mentioned A2D strains have very similar genome structures (Fig 4B); however, those two infections do not appear to have direct epidemiological linkage, as indicated by the full genome pairwise distance of 9.7% (SE 0.3%) between the two strains. For comparison, the full genome pairwise distance between unlinked individuals within this dataset was estimated by separately analyzing the five sequences within the CRF16_A2D sub-cluster, which yielded a median pairwise distance of 10.4% (range: 8.9–11.4%), and the 20 non-linked pure A1 genomes which yielded a median distance of 10.8% (range: 6.7–13.7%). Recombinant Breakpoint Analysis of the 33 recombinant forms observed in this study shows a large amount of diversity. The genome structures (Fig 4A) vary from a simple recombinant with 2 breakpoints to a very complex genome with 22 breakpoints. Other than the two strains that were closely related to CRF16_A2D, the rest of the recombinants were newly identified unique recombinant forms. Further confirmation of the genomic structure of the observed CRF16_A2D strains was obtained by performing additional breakpoint analysis of the entire CRF16 cluster using the SimPlot [37] analytical suite, with care given to include subtype D reference sequences used during the initial identification and analysis [38,39] of CRF16_A2D (S2 Fig).

### Sequence distance and epitope coverage by the KEA, CMDR, TZC, and mosaic vaccine inserts

Tables 1 and 2 show the median protein sequence distance and interquartile range (IQR) between the protein sequences of each immunogen expressed by the MVA and Ad26 vaccine inserts and either the pure A1 strains (Table 1) or the recombinant strains (Table 2) from the combined incident, prevalent, and higher-risk cohorts discussed thus far (the same strains shown in Figs 2 and 3). These distances provide a useful comparison point between the amino acid sequences of the observed strains in this epidemic and the candidate vaccines.

**Table 1:** Median distance between vaccine inserts and observed pure A1 strains.

Candidate vaccine	subtype	Gag % difference (IQR)	Pol % difference (IQR)	Env % difference (IQR)
**KEA**	A1	11.6% (10.2–12.4%)^a^	5.4% (4.9–6.0%)^a^	17.5% (16.0–19.9%)^a^
**CMDR**	CRF01_AE	13.3% (12.4–14.5%)	8.4% (7.8–9.0%)	31.3% (29.7–33.0%)
**TZC**	C	18.8% (18.1–20.0%)	9.9% (9.3–10.8%)	33.0% (31.4–34.7%)
**mosaic 1**	B	19.3% (18.2–20.0%)	9.9% (9.3–10.8%)	26.8% (24.9–28.0%)
**mosaic 2**	C	18.7% (18.0–20.1%)	9.9% (8.9–10.5%)	29.2% (27.4–30.6%)

^a^ Significantly closer sequence similarity vs. the other inserts listed (p < .01).

**Table 2:** Median distance between vaccine inserts and observed recombinant strains.

Candidate vaccine	subtype	Gag % difference (IQR)	Pol % difference (IQR)	Env % difference (IQR)
**KEA**	A1	16.1% (12.9–18.3%)	9.9% (6.6–12.0%)	21.8% (18.2–27.6%)^b^
**CMDR**	CRF01_AE	14.7% (13.3–16.5%)	10.2% (8.4–11.0%)	32.0% (30.6–34.9%)
**TZC**	C	17.5% (15.7–19.0%)	9.0% (8.2–9.9%)	33.5% (31.2–35.0%)
**mosaic 1**	B	16.3% (14.0–18.8%)	9.9% (9.0–10.4%)	26.0% (24.2–27.8%)^b^
**mosaic 2**	C	16.1% (14.9–18.0%)	8.7% (8.0–9.6%)	29.6% (27.4–32.1%)

^b^ Both Env inserts have significantly closer sequence similarity to the recombinants when compared to CMDR, TZC, or mosaic 2 (p < .01); however, KEA is not significantly closer to the recombinants than the mosaic 1 insert.

Higher values for sequence distances reflect larger numbers of differences between the sequences of the infecting strains and the vaccine under comparison. Distances between amino acid sequences are distinct from distances between nucleotide sequences and will vary among the different viral proteins. As a reference, Korber and colleagues [40] performed an amino acid sequence analysis of HIV-1 and calculated an intra-subtype difference of 17% (range: 4–30%) within Env and 8% (range: 2–15%) within Gag in subtypes A and B; and they found an inter-subtype difference of 25% (range: 20–36%) in Env and 17% (range: 15–22%) in Gag between subtypes A and B.

For the purpose of these distance comparisons, the mosaic vaccine inserts are treated separately; however in application, the mosaic 1 and 2 inserts would be administered simultaneously (1:1 ratio) within the bivalent mosaic vaccine. Among the pure A1 infections, the regionally matched KEA insert is significantly closer to Gag, Pol, and Env from the infecting strains as compared to the other vaccine inserts. While among the recombinant infections, only the Env from the KEA and mosaic 1 inserts show closer similarity to the infecting strains.

**Fig 5:**
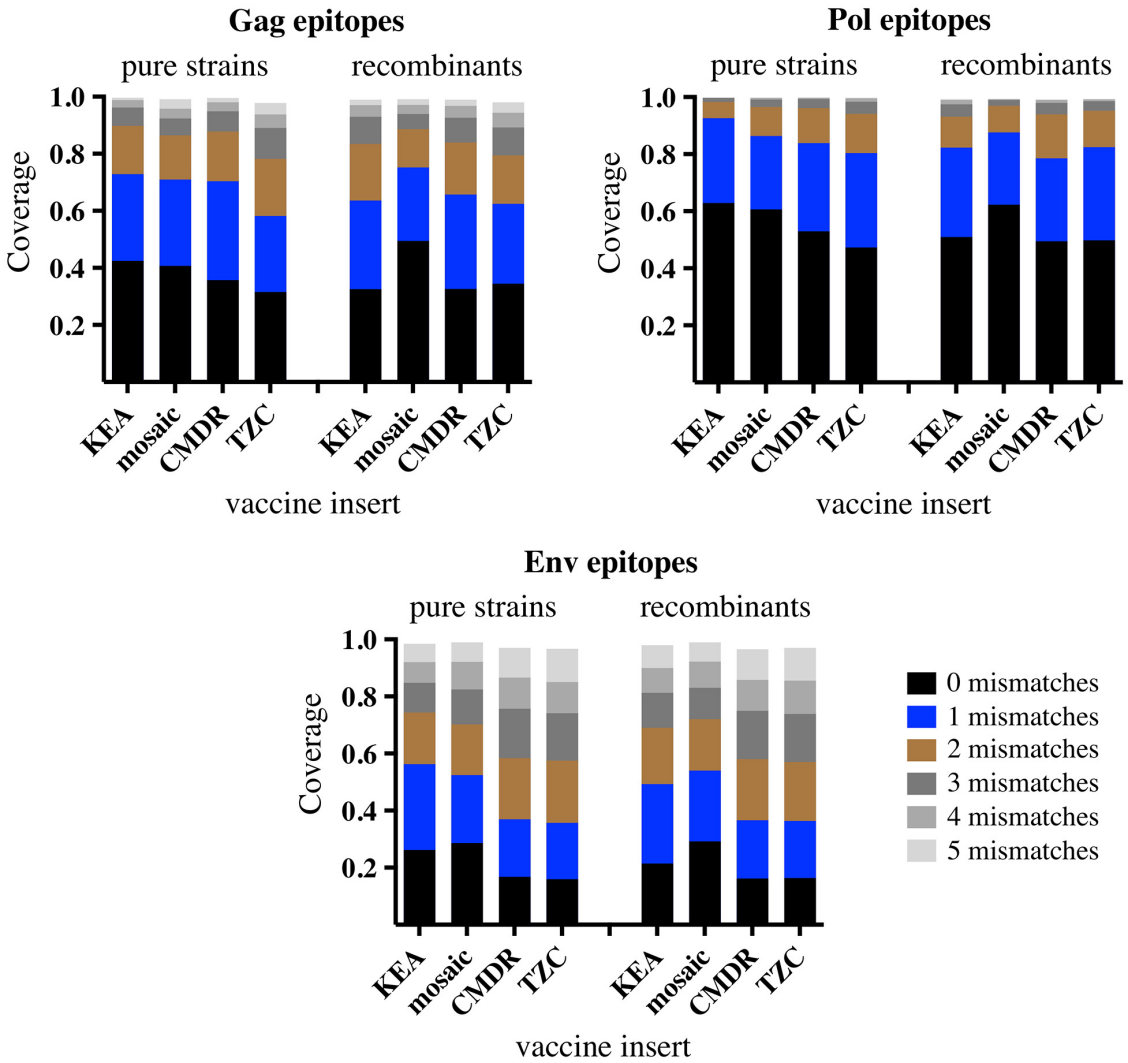
Vaccine coverage of potential T-cell epitopes from the observed infections. Inserts from the candidate vaccines were analyzed for the proportion of nonamers that each of their respective immunogens covered within the infecting strains from the incident, higher-risk, and prevalent infection studies. For each immunogen, the epitope coverage comparisons were divided into the same pure and recombinant strain subsets used to generate Tables 1 and 2. The colored sections of each bar denote the proportion of coverage attributable to a perfect match (black) or mismatched (blue to gray) nonamer as indicted by the figure legend.

Since the mosaic vaccine was designed to optimize the generation of T-cell epitopes, a comparison of the proportion of nonamers present in the infecting strains and overlapped by each vaccine was performed. The proportional coverage shown in Fig 5 represents the per-sequence average of nonamers present in the observed strains that were also present in the vaccine insert or inserts (as in the case of the bivalent mosaic vaccine). Further positional information is provided in Figures A-I in S1 File which include exact match nonamer positions, sequence logos [41] of the strain groups, and sequence positions of known CD8 epitopes for the viral protein regions used in these comparisons. By design [35], the mosaic vaccine (bivalent, containing subtypes B and C inserts) provides comparatively good nonamer coverage even within the recombinants; and while the Kenyan KEA insert provides better coverage among the pure strains, that advantage is lost once heterogeneity is introduced by the recombinants.

## Discussion

This study provides full genome HIV-1 sequencing from 58 incident infections in a Kenyan tea plantation community cohort from Kericho during 2003–2006. It is complementary to studies of prevalent infections among HIV-1 positive blood donations in southern Kenya (1999–2000) [8] and a combination of incident and prevalent infections in a higher-risk cohort from the Mombasa and Kilifi-Coast areas in 2006 [17]. Furthermore, the observed HIV-1 strains were compared to several candidate HIV-1 vaccine inserts in order to evaluate the potential suitability of those vaccines in East Africa.

In the prevalent infection (blood-bank) study, 60.9% of the strains were pure subtypes (23 A1, 1 C, and 1 D), while in the higher-risk cohort, 58.3% of the incident strains were pure subtypes (all A1). Here in this study, it was found that 43.1% of the strains from incident infections among members of a tea plantation cohort in Kenya’s southern Rift Valley are pure subtypes (21 A1, 3 C, and 1 G). Upon phylogenetic analysis, the interspersed pattern of sequences from each of these cohorts (see Figs 2 and 3), coupled with a sample collection timeframe spanning 1999 to 2006, suggests regional circulation of HIV-1 in Kenya and its immediate neighbors with no new introduction of A1 virus strains into the area. In contrast, the three pure subtype C incident infections, rare in Kenya, clustered with the single C virus found during the prevalent infection (blood bank) study in addition to subtype C strains from other regions (Botswana, Tanzania, Ethiopia, and India). This suggests the possibility that these C strains were introduced from outside of the Kenya region.

One of the more striking features of this epidemic is the proportion and complexity of inter-subtype recombinants. In the prevalent infection study (blood-bank from southern Kenya), recombinants constituted 38.8% of the strains; with A1D representing the majority at 14.6%, while A2D and A1C contributed equal proportions at 7.3% each, with the remaining strains consisting of A1A2D, A1CD, A1G and CD recombinants. Similarly, the higher-risk cohort (from Mombasa and Kilifi-Coast) had incident infections comprised of 41.7% inter-subtype recombinants, with A1 being the major parent subtype followed by subtypes D, C, and A2. In the incident infections of the current community cohort study, the proportion of recombinant strains was 56.9%. Once again, A1D recombinants represent the majority at 29.3%, followed by A1CD 8.6%, A1A2D 6.9%, and A1C at 5.2%.

In each of these studies, only strains related to CRF16_A2D or CRF21_A2D possessed genomic structures or phylogenetic relationships that would identify them as the progeny of known recombinant strains, the rest were unique recombinant forms (URF). Using currently available sequences, none of the URFs appeared to belong to a parental lineage of recombinants such as would be expected in the case of new infections involving recombinant transmission. Additionally, although the majority of the recombinants were A1D, there were no pure D strains observed within the incident infections of the community or higher-risk cohort studies; and only one pure D strain was observed in the blood-bank study. The apparent absence of parent strains containing subtype D, may be due to higher pathogenicity of subtype D infections [42,43], transmission events involving partners not sampled within these cohorts, or the inherit limitations of sample size. Nonetheless, the overall proportion of recombinant strains is substantial within these cohorts: blood-bank survey (38.8%), higher-risk cohort (41.7%), and community cohort (56.9%); which suggests that the process of HIV-1 recombination is ongoing within the Kenyan epidemic and may be leading to an epidemic dominated by unique recombinants.

The identification of the recombinants and the parent subtypes observed in these studies are in general agreement with previously published subtyping surveys of the Kenyan HIV-1 epidemic [44] [45] [46] [47] [15]. Any differences may be due to the quantitation of subtype D proportions by use of partial genome sequencing, which can misclassify recombinants as pure strains. Since subtype D recombinants are high in number, it is expected that some of them could be classified as D strains via partial genome sequencing. Regardless, the variety of recombinants and parent subtypes detected within each of these studies corroborates the overall strain composition observed during our full genome surveys.

With respect to vaccine development, viral genetic diversity within a prospective vaccine cohort can be regarded as an obstacle wherein epitopes of interest are made more difficult to target by vaccine-induced sequence specific immune responses. As such, the one Ad26- and four MVA- vectored vaccines under development for use in sub-Saharan Africa represent an interesting and useful evaluation point. While the KEA vaccine insert (isolated from a Kenyan infected with subtype A1 virus) would be the expected choice for vaccine deployment in Kenya, the heterogeneity within the recombinants largely nullifies the advantage conferred by subtype matching (Tables 1 and 2). In terms of sequence similarity to the recombinants (Table 2), both the KEA and mosaic-1 inserts produce Env that is closer to the recombinant strains than the Env from the CMDR, TZC, or mosaic-2 inserts; however, the KEA Env is not significantly closer to the recombinants when compared to the mosaic-1 Env. In that regard, simultaneous delivery of two different subtype vaccines (where one of them is a regional match) or a combination of subtype matched and mosaic vaccines, will be more likely to produce a robust immune response than a single subtype vaccine. Though it is unknown whether a combination of vectored isolate vaccines or mosaic vaccines would be efficacious in human populations, the genetic distances between the observed sequences and these vaccines are consistent with the distances reported in the successful SHIV study by Barouch et al [23]. Additionally, MVA vectored SIV vaccine studies using heterologous vaccine and challenge strains have shown that protective antibody responses to Env do not necessarily require matching of the vaccine immunogens to the eventual challenge [21]. In consideration of cellular responses, individually, neither of the mosaic inserts (subtypes B and C) provide remarkably greater epitope coverage than the CMDR or TZC inserts. However, in unison the bivalency of the mosaic design produces compensatory epitope coverage that allows for enhanced performance in both pure and recombinant strains, Fig 5.

As indicated by the growing list of recombinant forms in the Los Alamos HIV-1 database and the present data, the Kenyan epidemic will continue to evolve and produce a variety of unique recombinant strains. This diversity has the potential to reduce vaccine effectiveness. Without foreknowledge of the emerging recombinant strains, the application of a multivalent approach using both regionally tailored and global mosaic strategies may offer the greatest opportunity for a vaccine-based intervention in heterogeneous epidemics, such as the one described here. Regardless of the nature of the vaccine chosen, the genetic diversity within the Kenyan epidemic should prove to be a formidable challenge and will likely yield valuable information for use in the next generation of vaccines.

## Supporting Information

S1 Fig Extensive subtype G phylogenetic tree. Phylogenetic analysis of sample 06KE275457V6 (in red) and over 100 full-length subtype G and G recombinant sequences from the Los Alamos HIV-1 database and our own lab database; confirming the phylogenetic relationship of the lone G strain from the current incident cohort to Kenyan G strains identified during previous studies. The magnified subset shows the relevant bootstrap values. The scale bar indicates a genetic distance of 10%.
(PDF)

S2 Fig Genomic structure from Simplot analysis of CRF16_A2D cluster. The genomic structures of the two observed and three reference CRF16_A2D strains were calculated using a 300bp window and the three full-length subtype A2 sequences from the Los Alamos HIV database: 94CY017_41.AF286237, 97CDKTB48.AF286238, 01CM_1445MV.GU201516 and three full-length subtype D reference sequences: 94UG114.U88824, Z2Z6_Z2_CDC_Z34.M22639, NDK.M27323 used during initial characterization of the circulating recombinant form CRF16_A2D [38,39].
(PDF)

S1 File Proportional coverage by position for each vaccine and WebLogo graphs, CD8 epitope positions, and vaccine sequence alignments for Gag, Pol, and Env. Figures A-C are graph overlays showing the position of exact match nonamers from overlapping portions of each vaccine that are present in the pure (blue crossbars) or recombinant (solid red) strains for the respective protein regions. These are the same strain groups used for the Proportional Coverage calculations described in the main text. The amino acid alignment positions shown on the x-axis correspond to HXB2 positions: 1–500 for Gag, 156–595 for Pol (RT), and 1–680 for Env. Figures D-I present the HXB2 referenced sequence logo [41] for the same strain groups used in the Proportional Coverage and Positional Coverage calculations as well as the HXB2 referenced CD8 epitopes for those regions. The CD8 T-cell epitopes shown are current as of 2015-06-04 (hiv.lanl.gov/content/immunology) and have been observed in HIV-1 infected individuals. These high-resolution graphs were generated from the alignment used for the Positional Coverage graphs and will allow the reader to magnify and view fine details. The sequence shown on the epitope map portion of each graph, corresponds to the HXB2 reference sequence K03455. The CTL_CD8 epitope spreadsheet details the location, species, sequence, and HLA type (if known) for each of the epitopes shown.
(ZIP)
