# Supplementary material for: The Number and Complexity of Pure and Recombinant HIV-1 Strains Observed within Incident Infections during the HIV and Malaria Cohort Study Conducted in Kericho, Kenya, from 2003 to 2006
Source: PLoS One. 2015 Aug 19;10(8):e0135124. doi: 10.1371/journal.pone.0135124 (PMC4543584; doi:10.1371/journal.pone.0135124)
Supplement: S2 Fig — The genomic structures of the two observed and three reference CRF16_A2D strains were calculated using a 300bp window and the three full-length subtype A2 sequences from the Los Alamos HIV database: 94CY017_41.AF286237, 97CDKTB48.AF286238, 01CM_1445MV.GU201516 and three full-length subtype D reference sequences: 94UG114.U88824, Z2Z6_Z2_CDC_Z34.M22639, NDK.M27323 used during initial characterization of the circulating recombinant form CRF16_A2D [38,39]. (PDF) [file pone.0135124.s002.pdf]

S2 Figure. Genomic structure from Simplot analysis of CRF16_A2D branch.

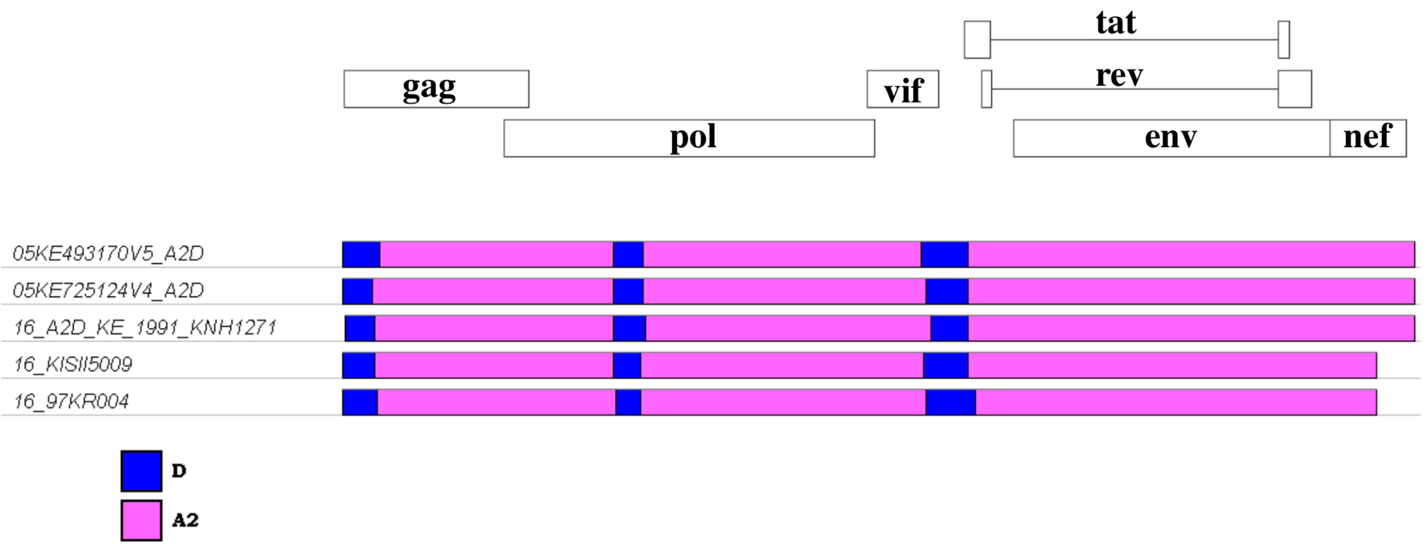

S2 Figure. Genomic structure from Simplot analysis of CRF16_A2D cluster. The genomic structures of the two observed and three reference CRF16_A2D strains were calculated using a 300bp window and the three full-length subtype A2 sequences from the Los Alamos HIV database: 94CY017_41.AF286237, 97CDKTB48.AF286238, 01CM_1445MV.GU201516 and three full-length subtype D reference sequences: 94UG114.U88824, Z2Z6_Z2_CDC_Z34.M22639, NDK.M27323 used during initial characterization of the circulating recombinant form CRF16_A2D.
