# Supplementary material for: The Number and Complexity of Pure and Recombinant HIV-1 Strains Observed within Incident Infections during the HIV and Malaria Cohort Study Conducted in Kericho, Kenya, from 2003 to 2006
Source: PLoS One. 2015 Aug 19;10(8):e0135124. doi: 10.1371/journal.pone.0135124 (PMC4543584; doi:10.1371/journal.pone.0135124)
Supplement: S1 File — Figures A-C are graph overlays showing the position of exact match nonamers from overlapping portions of each vaccine that are present in the pure (blue crossbars) or recombinant (solid red) strains for the respective protein regions. These are the same strain groups used for the Proportional Coverage calculations described in the main text. The amino acid alignment positions shown on the x-axis correspond to HXB2 positions: 1–500 for Gag, 156–595 for Pol (RT), and 1–680 for Env. Figures D-I present the HXB2 referenced sequence logo [41] for the same strain groups used in the Proportional Coverage and Positional Coverage calculations as well as the HXB2 referenced CD8 epitopes for those regions. The CD8 T-cell epitopes shown are current as of 2015-06-04 (hiv.lanl.gov/content/immunology) and have been observed in HIV-1 infected individuals. These high-resolution graphs were generated from the alignment used for the Positional Coverage graphs and will allow the reader to magnify and view fine details. The sequence shown on the epitope map portion of each graph, corresponds to the HXB2 reference sequence K03455. The CTL_CD8 epitope spreadsheet details the location, species, sequence, and HLA type (if known) for each of the epitopes shown. (ZIP) [file pone.0135124.s003.zip › S1_File/FigG3.pdf]

Recombinant Strains Gag part C

Figure S9c

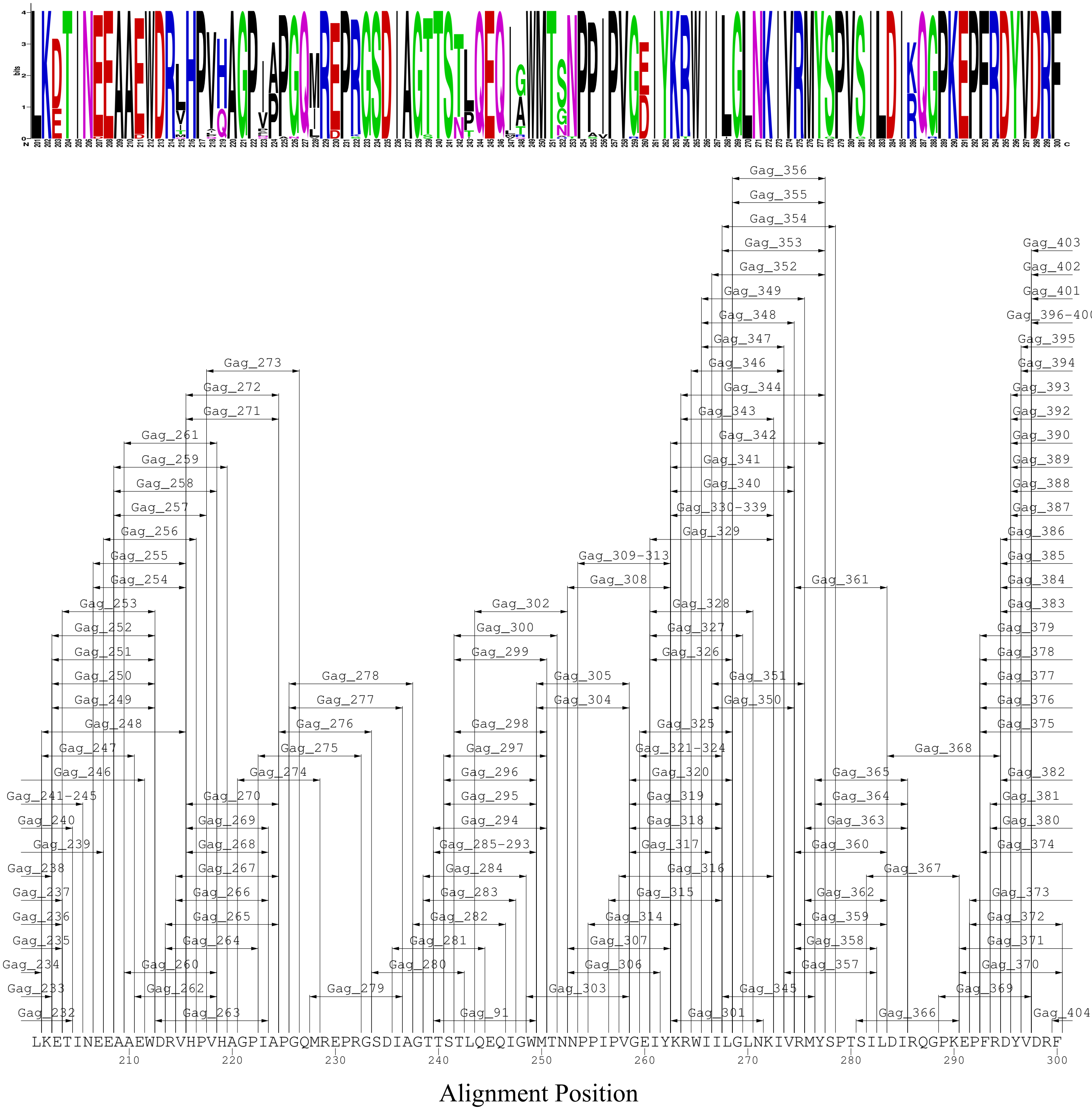


```
HXB2 LKETINEEAAEWDRVHPVHAGPIAPGQMREPRGSDIAGTTSTLQEQIGWMTNNPPIPVGEIYKRWIILGLNKIVRMYSPTSILDIRQGPKEPFRDYVDRF
Mos1 -------------------------------------------------------------------------------V--------------------
Mos2 --D-----------L-------V------------------N-----A---S-------D-------------------------K--------------
CMDR --------P--------------P-----------------------------------D-------------------V--------------------
TZC  --D-----------I-------T------------------------A---A---V-----------------------V-----K--------------
KEA  --------------L--AQ----P---I-D------------P----T-----------D-------------------V-----K--------------
```

Vaccine Alignment to HXB2 reference
